# The XBP1 Arm of the Unfolded Protein Response Induces Fibrogenic Activity in Hepatic Stellate Cells Through Autophagy

**DOI:** 10.1038/srep39342

**Published:** 2016-12-20

**Authors:** Rosa S. Kim, Daisuke Hasegawa, Nicolas Goossens, Takuma Tsuchida, Varinder Athwal, Xiaochen Sun, Christopher L. Robinson, Dipankar Bhattacharya, Hsin-I Chou, David Y. Zhang, Bryan C. Fuchs, Youngmin Lee, Yujin Hoshida, Scott L. Friedman

**Affiliations:** 1 Division of Liver Diseases, Department of Medicine, Liver Cancer Program, Tisch Cancer Institute, Graduate School of Biomedical Sciences, Icahn School of Medicine at Mount Sinai, New York, US; 2 Divisions of Gastroenterology and Hepatology, St. Marianna University School of Medicine, Kawasaki, Japan; 3 Divisions of Gastroenterology and Hepatology, Geneva University Hospital, Geneva, Switzerland; 4 Research Division, Mitsubishi Tanabe Pharma Corporation, Saitama, Japan; 5 Institute of Human Development, University of Manchester, United Kingdom; 6 Department of Surgery, Weill Cornell Medical College, New York, US; 7 Division of Surgical Oncology, Massachusetts General Hospital Cancer Center, Harvard Medical School, Boston, Massachusetts, US

## Abstract

Autophagy and the unfolded protein response (UPR) both promote activation of hepatic stellate cells (HSC), however the link between the two stimuli remains unclear. Here we have explored the role of X-box binding protein 1 (XBP1), one of three UPR effector pathways and sought to establish the interdependence between autophagy and the UPR during HSC activation. XBP1 induction accompanied both culture-based HSC activation and ER stress induced by tunicamycin. Ectopic overexpression of XBP1 induced collagen 1-alpha expression in HSCs, which was inhibited by knockdown of *ATG7*, a critical autophagy mediator. Genome-wide transcriptomic profiling indicated an upregulation of collagen synthesis pathways, but not of the transforming growth factor (TGF)-b pathway, a canonical fibrogenic driver, suggesting that XBP1 activates a specific subset of fibrogenesis pathways independent of TGF-β1. XBP1 target gene signatures were significantly induced in rodent liver fibrosis models (n = 3–5) and in human samples of non-alcoholic fatty liver disease (NAFLD) (n = 72–135). Thus, XBP1-mediated UPR contributes to fibrogenic HSC activation and is functionally linked to cellular autophagy.

Progressive liver fibrosis leads to cirrhosis, which affects 1% to 2% of the global population and accounts for more than one million deaths every year^1^,^2^. Liver injury from viral hepatitis, alcohol abuse, and non-alcoholic fatty liver diseases/non-alcoholic steatohepatitis (NAFLD/NASH), among others, promotes deposition of extracellular matrix (ECM) protein by myofibroblasts primarily derived from activated hepatic stellate cells (HSCs), the resident pericytes of the liver^3^. This fibrogenic phenotype of activated HSCs imposes endoplasmic reticulum (ER) stress to induce activation of the unfolded protein response (UPR). The UPR is a highly conserved stress-responsive signaling pathway that arises from three ER transmembrane proteins: activating transcription factor 6 (ATF6), protein kinase R-like ER kinase (PERK encoded by *EIF2AK3*), and inositol-requiring enzyme 1 (IRE1 encoded by *ERN1*)^4^. Each of the three proteins is linked to a distinct downstream cascade that together mitigate cellular stress and maintain homeostasis. While UPR in hepatocytes has been well studied, especially in the context of metabolic dysregulation^5^,^6^, its involvement in liver fibrogenesis and HSC activation is not as well-characterized^7^,^8^,^9^.

We previously demonstrated that ER stress signaling through one of the three ER transmembrane proteins, IRE1, promotes HSC activation and fibrogenesis by increasing the secretion of type I collagen. ER stress in HSCs is accompanied by enhanced autophagy, a housekeeping self-degradative process induced by the UPR in many cell types to eliminate misfolded or aggregated proteins and to maintain energy homeostasis^7^. Another ER stress effector pathway, PERK has also been recently implicated in hepatic fibrogenesis^8^. These studies suggest that ER signaling may represent a distinct and important class of fibrosis effectors.

Autophagy is a major cellular catabolic process that delivers proteins, organelles and other cellular components to the lysosome for degradation as well as fuel recycling, and we previously demonstrated that autophagy is required for stellate cell activation and hepatic fibrogenesis in mice^10^. While it has been demonstrated in various biological contexts that cellular stress activates a set of well-orchestrated processes including the UPR and autophagy to restore homeostasis or commit to apoptosis, the interplay across these pathways remains to be determined in the context of hepatic fibrosis.

In this study, we have characterized the UPR-driven fibrogenic cascade downstream of IRE1 that is mediated by its key downstream transcription factor, X-box transcriptional factor (XBP1)^11^. During ER stress, a chaperone protein BiP, dissociates from IRE1, leading to its trans-autophosphorylation and modification of the unspliced, inactive form of XBP1 transcript (uXBP1) into its active spliced form (sXBP1), which then induces the transcription of specific target genes^11^. We have explored the impact of both uXBP1 and sXBP1 on the fibrogenic phenotype of HSCs, characterized their global transcriptional consequences in human and mouse primary HSCs and HSC lines, and linked these XBP-1 associated responses to phenotypes in rodent models of liver injury as well as human fibrotic liver disease.

## Results

### Endogenous XBP1 is induced during culture activation of primary mouse HSCs

**Figure 1:**
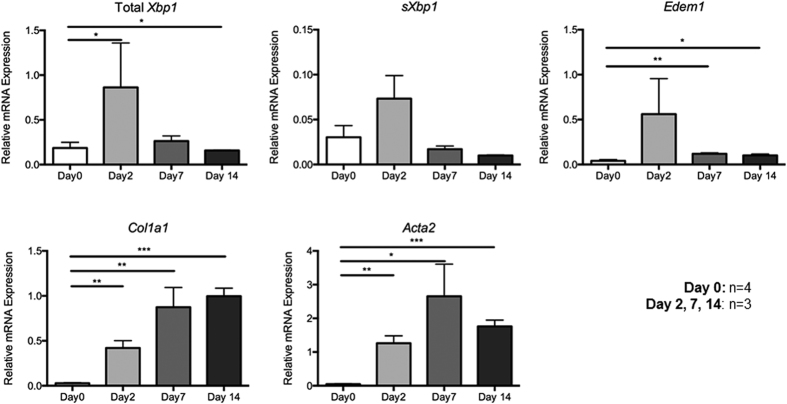
Early induction of XBP1 transcripts precedes collagen 1-alpha and alpha-smooth muscle actin gene expression during culture induced activation of primary murine HSCs. Cells from 3 month old C57Bl wildtype mice per sample were isolated as described in the Methods section, plated on uncoated tissue plastic in the presence of serum, and harvested for RNA and real time PCR immediately after isolation and after 2, 7 and 14 days. Relative quantification of transcripts for total and spliced, Xbp1, Edem1, collagen 1a1 and alpha smooth muscle actin (Acta2) is shown. Maximal induction of endogenous Xbp1 and sXbp1 are evident 2 days after culture induced activation, and precede more classic HSC activation markers (n = 3–4 for each group). *p < 0.05, **p < 0.01, ***p < 0.001.

We first determined the behavior of XBP1 during culture-induced HSC activation on a stiff plastic substratum, a well-established method to activate normal primary HSCs^12^. Primary HSCs isolated from 4 mice were cultured for 2, 7, and 14 days, and the expression of sXBP1 mRNA was quantified along with fibrosis-related genes by qPCR (Fig. 1). As expected, *COL1A1* (encoding collagen 1-alpha) and *ACTA2* (encoding alpha-smooth muscle actin [alpha-SMA]) mRNA expression increased during spontaneous HSC activation in culture (Fig. 1). Similarly, sXBP1 mRNA, as well as a known XBP1 target gene, *EDEM1*, were also induced, peaking at day 2 (Fig. 1), suggesting that the early phases of sXBP1 induction are temporally associated with fibrogenic gene induction.

### Chemical UPR activation by tunicamycin stimulates collagen 1-alpha expression, accompanied by XBP1 induction in a human HSC line

**Figure 2:**
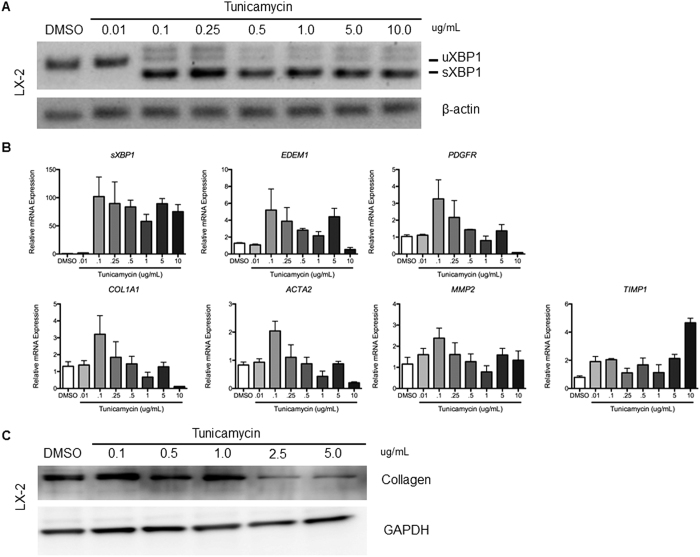
Coordinate induction of collagen 1-alpha and XBP1 seen following UPR activation from tunicamycin. Samples were collected after six-hour tunicamycin treatment of LX-2 cells at 0.01, 0.1, 0.25, 0.5, 1.0, 5.0, and 10.0 μg/mL, for (**A**) RT-PCR and assessed for XBP1 splicing, (**B**) real time PCR with key fibrogenic genes normalized to the housekeeping gene *GAPDH*, and expressed as fold change relative to DMSO controls, (**C**) Western blot of collagen 1-alpha. XBP1 gene expression correlates with that of key fibrogenic genes, and an upregulation of collagen 1-alpha protein is seen at low tunicamycin doses (0.1–1 μg/mL).

We previously demonstrated that chemical UPR activation by tunicamycin activates HSC through induction of autophagy^7^,^13^. To implicate XBP1 in this response, a human HSC line, LX-2, was treated with tunicamycin for 6 hours at 0.01, 0.1, 0.25, 0.5, and 1 μg/mL. The splicing of XBP1 was evident at 0.1 μg/mL, accompanied by increased collagen 1-alpha protein (Fig. 2A). Canonical fibrogenic genes, *COL1A1, ACTA2, PDGFRB, MMP2*, and *TIMP1* followed a mostly biphasic pattern of induction, with highest expression at 0.1 μg/mL, which was progressively attenuated at higher, and presumably more toxic doses (Fig. 2B), consistent with the knowledge that UPR-inducing drugs typically induce a level of ER stress that can be resolved, whereas high concentrations induce an ER burden that the UPR cannot overcome^14^, which is in line with our previous observation^7^. There was also upregulation of collagen 1-alpha protein at low tunicamycin doses (0.1–1 μg/mL) (Fig. 2C). These results collectively demonstrate that sXBP1 overexpression is associated with UPR-mediated HSC activation.

### XBP1 drives fibrogenic activation of human primary HSCs and HSC lines

**Figure 3:**
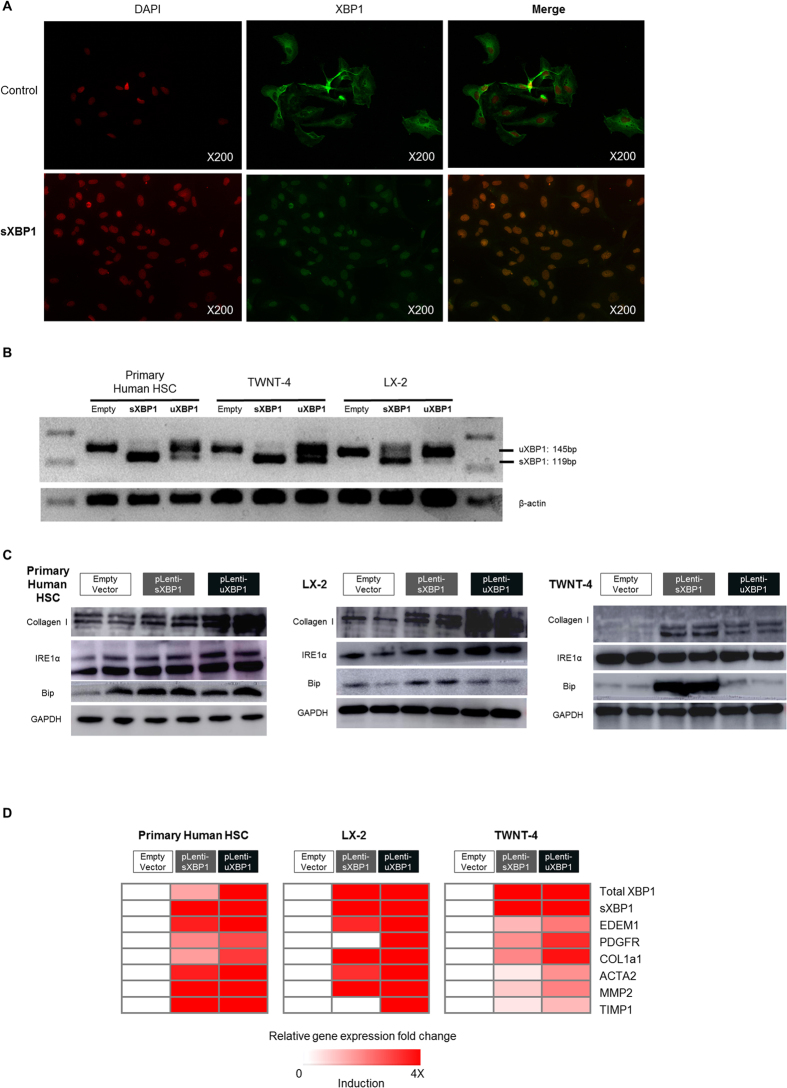
Virally expressed sXBP1 and uXBP1 induce collagen 1-alpha expression in human primary HSCs and human stellate cell lines (LX-2 and TWNT-4). Samples were collected 72 hours post infection for (**A**) immunofluorescence to visualize field-wide changes in XBP1 expression (**B**) RT-PCR of overexpressed XBP1 mRNA compared to empty vector. (**C**) Western blot for collagen 1-alpha, IRE1α, and BIP, (**D**) real time PCR to assess *COL1a* and other key fibrogenic transcripts, where gene expression values were normalized to the housekeeping gene *GAPDH*, and expressed as fold change relative to empty controls. Key fibrogenic genes and proteins were induced following XBP1 overexpression (n = 3–5 for each group).

To determine the causal role of XBP1 in fibrogenic HSC activation, we ectopically overexpressed both isoforms of XBP1 by lentiviral transduction in primary human HSCs as well as LX-2 and TWNT-4 cells, two well characterized human HSC lines. Successful transduction of XBP1 was confirmed by immunofluorescence (Fig. 3A), RT-PCR (Fig. 3B), and qPCR (Fig. 3D). Of note, efficient lentiviral expression of uXBP1 led to higher splicing and thus higher sXBP1 abundance in uXBP1-transduced cells, compared to sXBP1- transduced cells. Induction of the XBP1-target gene, *EDEM1* was also verified by qPCR (Fig. 3D). The fibrogenic genes/proteins, *COL1A1* (collagen 1-alpha), *ACTA2* (alpha-SMA), *PDGFRB, MMP2*, and *TIMP1*, were induced in association with increased sXBP1 (Fig. 3C and D). ER chaperone protein BiP was also induced by overexpression of XBP1, especially sXBP1, suggesting induction of severe ER stress by sXBP1. Together, these results from primary human HSCs as well as two human HSC cell lines indicate that XBP1 can induce fibrogenesis, a central feature of HSC activation, in cultured HSCs.

### Fibrogenic activation of HSCs by XBP1 requires autophagy

**Figure 4:**
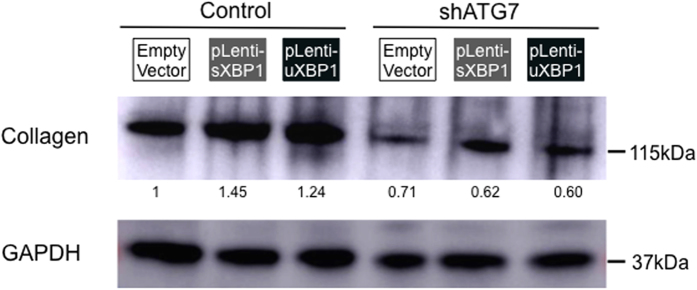
XBP1-mediated collagen production in HSCs requires autophagy. Autophagy was suppressed by transducing shATG7 in HSC cell line (JS-1) prior to the overexpression of XBP1, and Western blotting of collagen 1-alpha was performed. Relative intensity of each band normalized to GAPDH is shown under the blot (n = 1 for each group).

We previously reported that inhibition of autophagy reduces HSC activation^7^. To examine whether intact autophagy is required for XBP1 to stimulate HSC activation, autophagy was suppressed by knockdown of *ATG7* using a shRNA prior to the overexpression of XBP1. Diminished autophagy indeed abrogated the effect of XBP1 overexpression and subsequent increase in fibrogenic genes/proteins *COL1A1* (collagen 1-alpha) (Fig. 4). Thus, blocking autophagy in HSCs attenuates fibrogenic activity, which is not restored by overexpression of XBP1, indicating that HSC activation by XBP1 requires autophagy.

### Molecular pathways associated with XBP1-mediated UPR and fibrogenic activation

Molecular pathways modulated by XBP1 were explored using genome-wide transcriptomic profiling of the XBP1-overexpressing LX-2 and TWNT-4 human HSC lines. Confirming the biologic activity of exogenous XBP1 in these cells, both the UPR pathway gene set and the sXBP target gene set were significantly enriched in both sXBP1- and uXBP1- overexpressing cells (Supplementary Table 3). Interestingly, enriched molecular pathways were highly comparable between sXBP1 and uXBP1, presumably because a substantial fraction of ectopically expressed uXBP1 was spliced into sXBP1, as indicated in the RT-PCR (Fig. 3B). Protein metabolism-related pathways, (i.e., protein synthesis, resolution of misfolded proteins, and post-translational modification), were induced in response to XBP1 overexpression, consistent with up-regulation of the UPR, along with shifting of metabolic events including induction of Krebs cycle and respiratory electron transport chain genes. In contrast, inflammatory signaling associated with interferon and tumor necrosis factor (TNF)-alpha were suppressed. MYC target genes were significantly induced by XBP1, consistent with previous findings linking the UPR to oncogenic MYC pathway activation^15^,^16^.

Whereas collagen-related pathways were enriched in XBP1-overexpressing cells, transforming growth factor (TGF)-b signaling elements, which normally drive collagen transcription, were suppressed. Specifically *SMAD2* expression was unchanged (not shown), indicating that the XBP1-mediated UPR in HSCs is distinct from the alternative ER signaling cascade originating from PERK, as reported recently^8^.

### XBP1 is induced in HSCs in murine fibrosis models and advanced human liver disease

To verify the physiological relevance of the findings in cultured cells, we next sought evidence of XBP1 activation within the transcriptomic profiles of human and rodent fibrotic liver tissues. To specifically characterize UPR activation in HSCs from within whole liver mRNA profiles, a 100-gene HSC-specific sXBP1 target gene signature and 100-gene uXBP1 gene signature were established, defined as the top differentially expressed genes between the XBP1-overexpressing HSCs and control cells (Supplementary Table 4).

**Figure 5:**
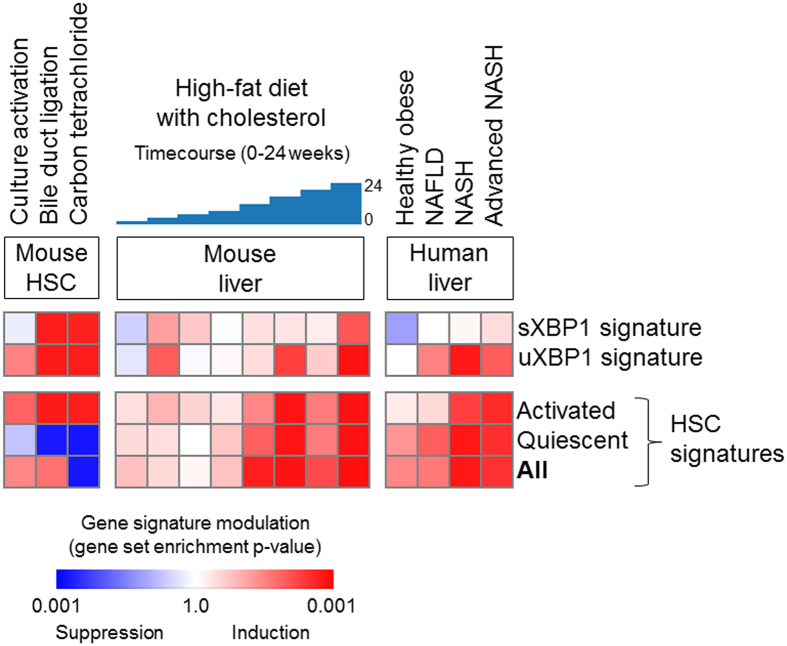
Significant co-induction of the XBP1 gene signatures and HSC gene signatures is seen with the progression of murine and human liver disease, specifically in (**A**) *ex vivo* culture-activated or *in vivo* activated mouse primary HSCs (NCBI Gene Expression Omnibus, GSE34640 with n = 3–5 per group), (**B**) liver tissues of time-course high-fat with cholesterol diet-treated NAFLD mouse model (GSE40481 with n = 3 per group), and (**C**) liver tissues of NAFLD patients according to the disease stages (simple steatosis, steatohepatitis, severe fibrosis (GSE49541 with n = 72, GSE61260 with n = 134), assessed by Gene Set Enrichment Analysis^33^. The heatmap indicates gene set enrichment p-values for each gene signature. The pattern of the XBP1 gene signature induction is tightly correlated with that of the HSC activation signature (p = 0.01 for sXBP1 signature and p < 0.001 for uXBP1 gene signature, Pearson correlation test).

These HSC specific XBP1 signatures were used to query a collection of two mouse and two human transcriptome datasets (Supplementary Table 5). First, both sXBP1 and uXBP1 signatures were strikingly induced in a mouse HSC-specific database of two commonly used experimental models of mouse liver fibrosis, carbon tetrachloride (CCl_4_) and bile duct ligation (BDL), implicating XBP1 activation in HSCs irrespective of the etiology (Fig. 5). Next, to link these findings to human disease, we interrogated two human datasets of NAFLD/NASH as well as that of a high fat diet-treated mouse model of NAFLD, confirming induction of our XBP1 signature that well-correlates with the HSC activation signature in both human disease and animal model. It should be noted that while we believe a subset of fibrogenesis pathways captured HSC activation signatures are included in our XBP1 signatures, there was very little gene overlap across them (Supplementary Figure 1).

## Discussion

Our findings indicate that XBP1, the downstream effector of IRE1, is induced during HSC activation in culture and *in vivo*, and we have functionally linked XBP1 to autophagy in this cell type. We have defined a set of genes unique to this branch of the UPR that is associated with HSC activation, and have utilized this gene set to establish the relevance of this pathway in HSCs to animal models and patients with liver diseases, in particular to NAFLD. While previously difficult to interrogate fibrosis progression in whole liver databases because HSCs consist of a minority of the overall liver volume, we have built upon our previous work^17^, to use our HSC activation signature to query publically available NAFLD databases, thus tracking HSC-specific changes in whole liver studies of disease progression. Our findings show a strong positive correlation between the HSC activation signature and HSC-specific XBP1 genes (p = 0.01 for sXBP1 signature and p < 0.001 for uXBP1 signature), reinforcing the hypothesis that XBP1-mediated ER stress plays an important role in HSC activation and progression of liver disease *in vivo*.

The UPR is a critical pathway to study in HSCs because once activated, these cells secrete increased collagen and other ECM molecules that challenge the cell’s ER homeostasis and protein-folding capacities. Two novel observations of this study merit comment in this context. First, we have specifically established sXBP1 as a key fibrogenesis effector. Whereas we previously described UPR-mediated changes in autophagy by knocking down IRE1^7^, it was unclear if IRE1’s activity was attributable to XBP1 activity or another IRE1-mediated response, such as selective cleaving of microRNAs to promote autophagy through c-Jun N-terminal kinase (JNK) activation^18^,^19^. Second, uXBP1 overexpression markedly induced type 1 collagen protein, a hallmark feature of activated HSCs, but we could not determine whether there was a substantial fraction of lentiviral-overexpressed uXBP1 that was spliced into sXBP1, or whether the effect is due to uXBP1 protein itself. In contrast to earlier studies, a recent study indicates that uXBP1 can effect cellular change specifically by modulating autophagy through FOXO1^20^.

Our data establish that the role of a URP effector, XBP1, in collagen 1-alpha production is mediated by autophagy, i.e., the UPR and autophagy pathways are orchestrated to promote fibrogenic activation of HSCs. The link between UPR and autophagy has been noted in other cell types^20^,^21^,^22^, but had not yet been implicated in HSC activation and collagen production^10^. Given the role of XBP1 in modulating glucose homeostasis and metabolic pathways in other cell types^23^,^24^, future studies to elucidate XBP1-mediated metabolic shifts in HSCs, and how they impact liver fibrogenesis merit evaluation.

The experiments in which sXBP1 was ectopically expressed have established its role in inducing UPR and fibrogenic HSC activation. Although sXBP1 levels may be supra-physiologic, the level of induction of *EDEM1*, a specific XBP1 target and surrogate for XBP1 activation, was comparable to effects of tunicamycin, a pharmacological UPR inducer. Nonetheless, because IRE1-XBP1 is one of three UPR branches that compensate for each other, it is difficult to definitely quantify its relative contribution to HSC activation compared to PERK and/or ATF6. Indeed, one recent study implicates PERK signaling in HSC activation as well^8^, whereas another reinforces the potential role of IRE1 in driving the UPR in fibrogenesis by myofibroblasts^25^.

While XBP1-mediated UPR stimulated collagen production in our system, it was not accompanied by an induction of the transforming growth factor (TGF)-b signaling pathway, as neither SMAD2 nor the TGF-β pathway was modulated in HSC lines overexpressing sXBP1. This TGF-β1-independent UPR mediated fibrogenesis contrasts with the aforementioned recent study that has implicated PERK-mediated UPR in fibrogenesis via the SMAD2/TGF-β pathway in HSCs^8^. Thus, while both XBP1- and PERK-mediated UPR seems to activate fibrogenesis, the mechanism by which this is achieved has distinct features. Because attempts to neutralize TGF-β therapeutically are limited by concern about loss of growth regulation and enhanced inflammation, pursuing the XBP1 branch of the UPR might circumvent this problem and emerge as a viable candidate for antifibrotic therapy by targeting collagen production distinct from the TGF-β pathway. Such effects would need to be restricted to HSCs through a cell targeting strategy, however.

In summary, our findings indicate that XBP1-mediated UPR activates HSCs through autophagy in a TGF-β-independent manner. Using bioinformatics approaches, this finding was linked to the pathogenesis of fibrosis in rodent models of liver injury and in patients with non-alcoholic steatohepatitis. The findings point towards future studies to determine whether the UPR in general, and sXBP1 in particular, represent potential therapeutic targets.

## Methods

### Cell culture

Two immortalized human HSC lines LX-2^26^ and TWNT-4^27^ cells, and immortalized mouse HSC line JS-1^28^ cells, were cultured in Dulbecco’s modification of Eagle’s medium (high glucose, sodium pyruvate-free) (Thermo Fisher Scientific) supplemented with 10% fetal bovine serum and penicillin/streptomycin at 37 °C and 5% CO_2_. Tunicamycin (Sigma) was dissolved in DMSO, added to the culture media, and incubated for 6 hours. The same concentration of DMSO was maintained in the culture media of control samples.

### Reverse transcriptase quantitative PCR (qPCR)

Total RNA was extracted from adherent cells using the RNeasy Mini kit (Qiagen). Equimolar concentrations of RNA were converted to cDNA (Clontech), and qPCR was performed using SYBR green qPCR master kit (Bio-Rad) and Lightcycler 480 (Roche) according to the manufacturer’s instruction. RNA expression level normalized to housekeeping gene, *GAPDH*, and respective control sample was calculated by delta-delta Ct method. Human and mouse PCR primer sequences used are listed in Supplementary Table 129.

### Lentiviral transduction of XBP1 expression

Viral coat plasmid (pCMV VSV-G), viral packaging plasmid (pCMV-dR8.2 dvpr) and lentiviral vector (pLenti-C-Myc-DDK) subcloned with open reading frame of either sXBP1 or uXBP1 (Origene) were transfected into 50% confluent 293 T cells using the X-treme gene HP DNA Transfection reagent (Roche). Empty vector was used for control cells. Virus supernatant was collected after 48 and 72 hours, passed through a 0.2 μm filter to remove residual 293 T cells, and stored at 4 °C until use. Viral supernatant was concentrated using Lenti-X concentrator (Clontech), and added to target cells. After 6 hours, media was changed to fresh media. Cells were cultured for 96 hours, and harvested for subsequent analysis.

### *ATG7* gene knockdown by short hairpin (sh) RNA

Small hairpin (sense-loop-antisense) RNAs for *Atg*7 cloned into lentiviral vectors as previously described^10^,^30^,^31^ were used.

### Primary mouse and human HSC isolation and culture

Livers from 6 week-old male C57BL/6 mice were perfused with 30 mL HBSS (without calcium and magnesium), followed by 30 mL HBSS with 0.2% pronase, followed by 0.05% collagenase and 0.05% pronase. Subsequently, livers were excised, mechanically disrupted, and further digested *ex vivo* with 0.05% pronase and 0.05% collagenase in HBSS for 30 minutes at 37 °C^32^. The resulting cell suspension was filtered through a 70 μm filter and spun at 1,800 rpm for 10 minutes, and washed three times in PBS. Cell fractions buoyant in 31.5% Percoll were isolated after spinning for 30 minutes at 2,400 rpm, filtered, and washed twice with DMEM. Cells were pelleted at 1,800 rpm for 6 minutes and either collected immediately, or plated on flasks for culture. Human primary HSCs were isolated from discarded remnants of surgically resected human livers that lacked patient identifiers. All experimental protocols were approved by the Mount Sinai Institutional Review Board and IACUC committee. Animal experiments were performed following Guide for the Care and Use of Laboratory Animals (National Academy of Sciences) and the Mount Sinai institutional guidelines.

### Cell Immunofluorescence

Adherent LX-2 and TWNT-4 cells were fixed with ice-cold 4% PFA for 15 minutes, permeabilized with 0.2% Triton X-100 in PBS for 20 minutes, rinsed three times with PBS, and blocked with 1% BSA/PBS for one hour. Staining was performed with primary antibody in 1% BSA solution at 4 °C overnight; primary antibody detection was performed with Alexa Fluor 488-conjugated goat anti-mouse IgG or Alexa Fluor 647-conjugated goat anti-rabbit. After PBS wash, the slides were mounted by DAPI fluoromount G (Southern Biotech) and imaged using Nikon Eclipse Ni-U microscope (Nikon). Antibodies used are listed in Supplementary Table 2.

### Western blot

Adherent LX-2, TWNT-4 and primary HSCs were lysed in RIPA (10 mM Tris-Cl, 1 mM EDTA, 0.5 mM EGTA, 1% Triton X-100, 0.1% sodium deoxycholate, 0.1% SDS, 140 mM NaCl) and was resolved in 4–12% precast Bis-Tris (NuPage Novex) polyacrylamide gels, transferred onto PVDF membranes, blocked with 5% nonfat milk in PBST, and blotted according to the antibody combinations listed in Supplementary Table 2.

### Transcriptome analysis

Two hundred ng of total RNA samples from LX-2 and TWNT-4 cells were assayed on the Human HT-12 v4 beadchip (Illumina) following manufacturer’s protocol. Scanned raw data were normalized by cubic spline algorithm implemented in Illumina normalizer module of GenePattern genomic analysis toolkit (www.broadinstitute.org/genepattern). Dataset is available at NCBI Gene Expression Omnibus (GEO) database (accession number: GSE78070). Additionally, publicly available transcriptome datasets of human and mouse models of non-alcoholic steatohepatitis/non-alcoholic fatty liver diseases (NASH/NAFLD) were obtained from GEO (Supplementary Table 5), and assessed for induction of our XBP1 target gene signature.

### Bioinformatics and statistical analysis

Continuous variables are presented as median and IQR. Inter-group difference of continuous variables was tested by Wilcoxon rank-sum test. Correlation between continuous variables was assessed by Spearman rank correlation test. Modulated molecular pathways were determined by surveying a comprehensive molecular pathway gene set collection (Molecular Signature Database, www.broadinstitute.org/msigdb) using Gene Set Enrichment Analysis (GSEA)^33^. The XBP1 target gene signature was defined as differentially expressed genes by random permutation *t*-test after eliminating less variable genes across the samples at coefficient of variation (CV) cut-off value of 0.5. Induction of the gene signature in the public human and mouse datasets was assessed by GSEA and nearest template prediction (NTP) algorithm^34^. Two-tailed p < 0.05 or false discovery rate (FDR)  < 0.05 were regarded as statistically significant. All analyses were performed using GenePattern or R statistical language (www.r-project.org).

## Additional Information

**How to cite this article**: Kim, R. S. *et al*. The XBP1 Arm of the Unfolded Protein Response Induces Fibrogenic Activity in Hepatic Stellate Cells Through Autophagy. *Sci. Rep.* **6**, 39342; doi: 10.1038/srep39342 (2016).

**Publisher's note:** Springer Nature remains neutral with regard to jurisdictional claims in published maps and institutional affiliations.

## Supplementary Material

Supplementary Information

## References

[b1] Friedman S. L. Evolving challenges in hepatic fibrosis. Nat Rev Gastroenterol Hepatol 7, 425–36 (2010). 20585339 10.1038/nrgastro.2010.97

[b2] Lozano R. . Global and regional mortality from 235 causes of death for 20 age groups in 1990 and 2010: a systematic analysis for the Global Burden of Disease Study 2010. Lancet 380, 2095–128 (2012). 23245604 10.1016/S0140-6736(12)61728-0 PMC10790329

[b3] Friedman S. L. Hepatic stellate cells: protean, multifunctional, and enigmatic cells of the liver. Physiol Rev 88, 125–72 (2008). 18195085 10.1152/physrev.00013.2007 PMC2888531

[b4] Walter P. & Ron D. The unfolded protein response: from stress pathway to homeostatic regulation. Science 334, 1081–6 (2011). 22116877 10.1126/science.1209038

[b5] Liu X. . Hepatocyte X-box binding protein 1 deficiency increases liver injury in mice fed a high-fat/sugar diet. Am J Physiol Gastrointest Liver Physiol 309, G965–74 (2015). 26472223 10.1152/ajpgi.00132.2015 PMC4683298

[b6] Han C. Y., Lim S. W., Koo J. H., Kim W. & Kim S. G. PHLDA3 overexpression in hepatocytes by endoplasmic reticulum stress via IRE1-Xbp1s pathway expedites liver injury. Gut (2015). 10.1136/gutjnl-2014-308506 PMC4975835 25966993

[b7] Hernandez-Gea V. . Endoplasmic reticulum stress induces fibrogenic activity in hepatic stellate cells through autophagy. J Hepatol 59, 98–104 (2013). 23485523 10.1016/j.jhep.2013.02.016 PMC3686909

[b8] Koo J. H., Lee H. J., Kim W. & Kim S. G. Endoplasmic Reticulum Stress in Hepatic Stellate Cells Promotes Liver Fibrosis via PERK-Mediated Degradation of HNRNPA1 and Up-regulation of SMAD2. Gastroenterology 150, 181–193 e8 (2016). 26435271 10.1053/j.gastro.2015.09.039

[b9] Hasegawa D. & Friedman W. M. SL. Stellate cells and hepatic fibrosis, 41–62 (Elsevier, 2015).

[b10] Hernandez-Gea V. . Autophagy releases lipid that promotes fibrogenesis by activated hepatic stellate cells in mice and in human tissues. Gastroenterology 142, 938–46 (2012). 22240484 10.1053/j.gastro.2011.12.044 PMC3439519

[b11] Gardner B. M. & Walter P. Unfolded proteins are Ire1-activating ligands that directly induce the unfolded protein response. Science 333, 1891–4 (2011). 21852455 10.1126/science.1209126 PMC3202989

[b12] De Minicis S. . Gene expression profiles during hepatic stellate cell activation in culture and *in vivo*. Gastroenterology 132, 1937–46 (2007). 17484886 10.1053/j.gastro.2007.02.033

[b13] Lehrman M. A., Zhu X. Y. & Khounlo S. Amplification and molecular cloning of the hamster tunicamycin-sensitive N-acetylglucosamine-1-phosphate transferase gene. The hamster and yeast enzymes share a common peptide sequence. J Biol Chem 263, 19796–803 (1988). 2848842

[b14] Vacaru A. M. . Molecularly defined unfolded protein response subclasses have distinct correlations with fatty liver disease in zebrafish. Dis Model Mech 7, 823–35 (2014). 24973751 10.1242/dmm.014472 PMC4073272

[b15] Hart L. S. . ER stress-mediated autophagy promotes Myc-dependent transformation and tumor growth. J Clin Invest 122, 4621–34 (2012). 23143306 10.1172/JCI62973 PMC3533536

[b16] Zhang D. Y. & Friedman S. L. Fibrosis-dependent mechanisms of hepatocarcinogenesis. Hepatology 56, 769–75 (2012). 22378017 10.1002/hep.25670 PMC4087159

[b17] Zhang D. Y. . A hepatic stellate cell gene expression signature associated with outcomes in hepatitis C cirrhosis and hepatocellular carcinoma after curative resection. Gut (2015). 10.1136/gutjnl-2015-309655 PMC4848165 26045137

[b18] Urano F. . Coupling of stress in the ER to activation of JNK protein kinases by transmembrane protein kinase IRE1. Science 287, 664–6 (2000). 10650002 10.1126/science.287.5453.664

[b19] Ogata M. . Autophagy is activated for cell survival after endoplasmic reticulum stress. Mol Cell Biol 26, 9220–31 (2006). 17030611 10.1128/MCB.01453-06 PMC1698520

[b20] Zhao Y. . XBP-1u suppresses autophagy by promoting the degradation of FoxO1 in cancer cells. Cell Res 23, 491–507 (2013). 23277279 10.1038/cr.2013.2 PMC3616429

[b21] Senft D. & Ronai Z. A. UPR, autophagy, and mitochondria crosstalk underlies the ER stress response. Trends Biochem Sci 40, 141–8 (2015). 25656104 10.1016/j.tibs.2015.01.002 PMC4340752

[b22] Adolph T. E. . Paneth cells as a site of origin for intestinal inflammation. Nature 503, 272–6 (2013). 24089213 10.1038/nature12599 PMC3862182

[b23] Lee A. H., Scapa E. F., Cohen D. E. & Glimcher L. H. Regulation of hepatic lipogenesis by the transcription factor XBP1. Science 320, 1492–6 (2008). 18556558 10.1126/science.1158042 PMC3620093

[b24] Yang L. . METABOLISM. S-Nitrosylation links obesity-associated inflammation to endoplasmic reticulum dysfunction. Science 349, 500–6 (2015). 26228140 10.1126/science.aaa0079 PMC4573582

[b25] Heindryckx F. . Endoplasmic reticulum stress enhances fibrosis through IRE1alpha-mediated degradation of miR-150 and XBP-1 splicing. EMBO Mol Med 8, 729–44 (2016). 27226027 10.15252/emmm.201505925 PMC4931288

[b26] Xu L. . Human hepatic stellate cell lines, LX-1 and LX-2: new tools for analysis of hepatic fibrosis. Gut 54, 142–51 (2005). 15591520 10.1136/gut.2004.042127 PMC1774377

[b27] Shibata N. . Establishment of an immortalized human hepatic stellate cell line to develop antifibrotic therapies. Cell Transplant 12, 499–507 (2003). 12953924 10.3727/000000003108747064

[b28] Guo J. . Functional linkage of cirrhosis-predictive single nucleotide polymorphisms of Toll-like receptor 4 to hepatic stellate cell responses. Hepatology 49, 960–8 (2009). 19085953 10.1002/hep.22697 PMC2891538

[b29] Hasegawa D. . Epithelial Xbp1 is required for cellular proliferation and differentiation during mammary gland development. Mol Cell Biol 35, 1543–56 (2015). 25713103 10.1128/MCB.00136-15 PMC4387219

[b30] Singh R. . Autophagy regulates lipid metabolism. Nature 458, 1131–5 (2009). 19339967 10.1038/nature07976 PMC2676208

[b31] Singh R. . Autophagy regulates adipose mass and differentiation in mice. J Clin Invest 119, 3329–39 (2009). 19855132 10.1172/JCI39228 PMC2769174

[b32] Friedman S. L. . Isolated hepatic lipocytes and Kupffer cells from normal human liver: morphological and functional characteristics in primary culture. Hepatology 15, 234–43 (1992). 1735526 10.1002/hep.1840150211

[b33] Subramanian A. . Gene set enrichment analysis: a knowledge-based approach for interpreting genome-wide expression profiles. Proc Natl Acad Sci USA 102, 15545–50 (2005). 16199517 10.1073/pnas.0506580102 PMC1239896

[b34] Hoshida Y. Nearest Template Prediction: A Single-Sample-Based Flexible Class Prediction with Confidence Assessment. PLoS One 5, e15543 (2010). 21124904 10.1371/journal.pone.0015543 PMC2990751

