# Supplementary material for: The XBP1 Arm of the Unfolded Protein Response Induces Fibrogenic Activity in Hepatic Stellate Cells Through Autophagy
Source: Sci Rep. 2016 Dec 20;6:39342. doi: 10.1038/srep39342 (PMC5172197; doi:10.1038/srep39342)
Supplement: Supplementary Information [file srep39342-s1.pdf]

Supporting Information

The XBP1 Arm of the Unfolded Protein Response Induces Fibrogenic Activity in Hepatic Stellate Cells Through Autophagy

Rosa S. Kim, Daisuke Hasegawa, Nicolas Goossens, Takuma Tsuchida, Varinder Athwal, Xiaochen Sun, Christopher L. Robinson, Dipankar Bhattacharya, Hsin-I Chou, David Y. Zhang, Bryan C. Fuchs, Youngmin Lee, Yujin Hoshida, Scott L. Friedman

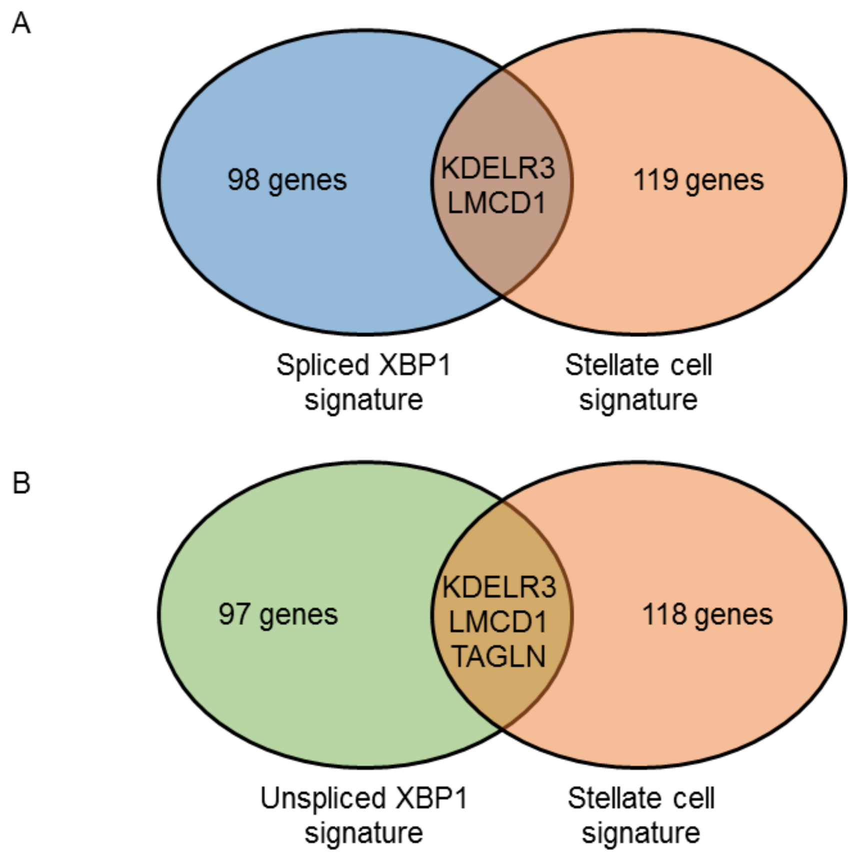

Supplementary Figure 1
Overlapped genes between the XBP1 target gene signatures (Supplementary Table 4) and the 122-gene HSC gene signature (Zhang et al., 20016).
(**A**) Overlap between overexpressed genes by sXBP1 and the HSC gene signature.
(**B**) Overlap between overexpressed genes by uXBP1 and the HSC gene signature.

Supplementary Table 1

Primer sequences used for qPCR.

Entrez Gene ID	Gene Symbol	Species	Sequence 5' to 3'	Scale (µmole)
2597	GAPDH-F	human	CAATGACCCCTTCATTGACC	0.025
2597	GAPDH-R	human	GATCTCGCTCCTGGAAGATG	0.025
1277	Col1-F	human	GGCTTCCCTGGTCTTCCTGG	0.025
1277	Col1-R	human	CCAGGGGGTCCAGCCAAT	0.025
59	ASMA-F	human	AGGCACCCCTGAACCCCAA	0.025
59	ASMA-R	human	CAGCACCGCCTGGATAGCC	0.025
5159	PDGFRB-F	human	CCAGAAGCCATCAGCAGCAAG	0.025
5159	PDGFRB-R	human	AGGCCCTGAGAGATCTGTGG	0.025
7076	TIMP1-F	human	TGACATCCGGTTCGTCTACA	0.025
7076	TIMP1-R	human	TGATGTGCAAGAGTCCATCC	0.025
7077	TIMP2-F	human	AAGCGGTCAGTGAGAAGGAA	0.025
7077	TIMP2-R	human	GGGGGCCGTGTAGATAAACT	0.025
4313	MMP2-F	human	CCCCAAGCTCATCGCAGAT	0.025
4313	MMP2-R	human	GGTCCACGACGGCATCC	0.025
7494	XBP1 total-F	human	CTGAATCTGAAGAGTCAATACCGCCAGAAT	0.025
7494	XBP1 total-R	human	AGGAGTTAAGACAGCGCTTGGGGATGGAT	0.025
7494	XBP1s 436-F	human	AACCAGGAGTTAAGACAGCGCTT	0.025
7494	XBP1s 569-R	human	CTGCACCCTCTGCGGACT	0.025
9695	EDEM-F	human	TTGACAAAGATTCCACCGTCC	0.025
9695	EDEM-R	human	TGTGAGCAGAAAGGAGGCTTC	0.025
22433	XBP1 total-F	mouse	CCTGAGCCCGGAGGAGAA	0.025
22433	XBP1 total-R	mouse	CTCGAGCAGTCTGCGCTG	0.025
22433	XBP1s-F	mouse	ACACGCTTGGGAATGGACAC	0.025
22433	XBP1s-R	mouse	CCATGGGAAGATGTTCTGGG	0.025
192193	EDEM-F	mouse	AAGCCCTCTGGAACTTGCG	0.025
192193	EDEM-R	mouse	AACCCAATGGCCTGTCTGG	0.025
11433	GAPDH-F	mouse	CCTGCCAAGTATGATGAC	0.025
11433	GAPDH-R	mouse	GGAGTTGCTGTTGAAGTC	0.025
12842	Col1a1-F	mouse	GTCCCTGAAGTCAGCTGCATA	0.025
12842	Col1a1-R	mouse	TGGGACAGTCCAGTTCTTCAT	0.025
11475	ASMA-F	mouse	TCCTCCCTGGAGAAGAGCTAC	0.025
11475	ASMA-R	mouse	TATGGTGGTTTCGTGGATGC	0.025

Supplementary Table 2

Antibodies used for Western blotting and immunostaining.

Protein	Dilution	Vendor	Catalog number	Protein Accession #
XBP1	1:200	Santa Cruz	M-186	Q9R1S4
Collagen	1:5000	Rockland	600-401-103	P02453
BIP	1:1000	Cell Signaling	3177	P11021
CHOP	1:1000	Cell Signaling	2895	P35638
pIRE1	1:1000	Abcam	ab48187	O75460
GAPDH	1:5000	Abcam	ab9482	P04406

Supplementary Table 3

Significantly molecular pathways modulated by sXBP1 or uXBP1 overexpression in human HSC lines (FDR<0.05, top 20 gene sets).
(Gene Set Enrichment Analysis)
Click gene set name for detailed annotation.

Induced in	Gene set database	Gene set	NES	p-value	FDR
sXBP1	Hallmark	UNFOLDED_PROTEIN_RESPONSE	2.44	<0.001	<0.001
	(curated transcriptional targets)	MTORC1_SIGNALING	2.37	<0.001	<0.001
		OXIDATIVE_PHOSPHORYLATION	2.29	<0.001	<0.001
		MYC_TARGETS_V1	2.21	<0.001	<0.001
		PROTEIN_SECRETION	2.18	<0.001	<0.001
		MYC_TARGETS_V2	1.84	<0.001	0.001
		FATTY_ACID_METABOLISM	1.83	<0.001	<0.001
		GLYCOLYSIS	1.74	<0.001	0.002
		ADIPOGENESIS	1.58	<0.001	0.008
		REACTIVE_OXIGEN_SPECIES_PATHWAY	1.58	0.008	0.008
		XENOBIOTIC_METABOLISM	1.57	<0.001	0.007
		ANDROGEN_RESPONSE	1.48	0.006	0.017
		CHOLESTEROL_HOMEOSTASIS	1.44	0.018	0.024
		DNA_REPAIR	1.42	0.010	0.027
		HYPOXIA	1.41	0.004	0.029
	Reactome	ASPARAGINE_N_LINKED_GLYCOSYLATION	2.76	<0.001	<0.001
	(curated pathway members)	METABOLISM_OF_PROTEINS	2.53	<0.001	<0.001
		POST_TRANSLATIONAL_PROTEIN_MODIFICATION	2.50	<0.001	<0.001
		ACTIVATION_OF_CHAPERONE_GENES_BY_XBP1S	2.39	<0.001	<0.001
		TRANSLATION	2.36	<0.001	<0.001
		UNFOLDED_PROTEIN_RESPONSE	2.36	<0.001	<0.001
		DIABETES_PATHWAYS	2.34	<0.001	<0.001
		BIOSYNTHESIS_OF_THE_N_GLYCAN_PRECURSOR_DOLICHOL_LIPID_LINKED_OLIGOSACCHARIDE_LLO_AND_TRANSFER_TO_A_NASCENT_PROTEIN	2.28	<0.001	<0.001
		SRP_DEPENDENT_COTRANSLATIONAL_PROTEIN_TARGETING_TO_MEMBRANE	2.26	<0.001	<0.001
		TRANSPORT_TO_THE_GOLGI_AND_SUBSEQUENT_MODIFICATION	2.22	<0.001	<0.001
		MITOCHONDRIAL_PROTEIN_IMPORT	2.21	<0.001	<0.001
		MEMBRANE_TRAFFICKING	2.08	<0.001	<0.001
		TCA_CYCLE_AND_RESPIRATORY_ELECTRON_TRANSPORT	1.96	<0.001	0.002

		RESPIRATORY_ELECTRON_TRANSPORT_ATP_SYNTHESIS_BY_CHEMIOSMOTIC_COUPLING_AND_HEAT_PRODUCTION_BY_UNCOUPLING_PROTEINS_	1.94	<0.001	0.002
		INCRETIN_SYNTHESIS_SECRETION_AND_INACTIVATION	1.91	<0.001	0.004
		BRANCHED_CHAIN_AMINO_ACID_CATABOLISM	1.90	<0.001	0.004
		P53_INDEPENDENT_G1_S_DNA_DAMAGE_CHECKPOINT	1.85	<0.001	0.008
		SYNTHESIS_SECRETION_AND_INACTIVATION_OF_GLP1	1.83	0.002	0.011
		CDK_MEDIATED_PHOSPHORYLATION_AND_REMOVAL_OF_CDC6	1.82	<0.001	0.013
		RESPIRATORY_ELECTRON_TRANSPORT	1.82	<0.001	0.013
	Transcription factor targets*	XBP1_01	2.09	<0.001	<0.001
		GABP_B	1.91	<0.001	0.001
		ATF6_01	1.87	<0.001	0.001
		ELK1_02	1.82	<0.001	0.002
		NRF2_01	1.82	<0.001	0.002
		HTF_01	1.77	<0.001	0.002
		TGANNYRGCA_TCF11MAFG_01	1.63	<0.001	0.009
		ATF_01	1.58	<0.001	0.013
		E4F1_Q6	1.48	<0.001	0.031
		CETS1P54_01	1.44	<0.001	0.047
	Signaling pathway targets** (MSigDB, C6 category)	CAMP_UP.V1_UP	2.02	<0.001	<0.001
		MTOR_UP.N4.V1_UP	1.69	<0.001	0.008
		EIF4E_UP	1.58	0.002	0.024
		NFE2L2.V2	1.54	<0.001	0.028
		CSR_LATE_UP.V1_UP	1.41	0.009	0.114
		ERB2_UP.V1_UP	1.39	0.006	0.103
		MYC_UP.V1_UP	1.37	0.018	0.105
		LTE2_UP.V1_UP	1.33	0.021	0.142
		PIGF_UP.V1_UP	1.32	0.023	0.147
		PDGF_UP.V1_UP	1.29	0.048	0.151
Control (vs. sXBP1)	Hallmark (curated transcriptional targets)	INTERFERON_ALPHA_RESPONSE	-2.56	<0.001	<0.001
		INTERFERON_GAMMA_RESPONSE	-2.38	<0.001	<0.001
		TNFA_SIGNALING_VIA_NFKB	-2.22	<0.001	<0.001
		TGF_BETA_SIGNALING	-2.10	<0.001	<0.001
		APOPTOSIS	-2.04	<0.001	<0.001
		P53_PATHWAY	-2.00	<0.001	<0.001
		KRAS_SIGNALING_UP	-1.79	<0.001	0.001
		IL6_JAK_STAT3_SIGNALING	-1.73	0.002	0.002

	INFLAMMATORY_RESPONSE	-1.71	<0.001	0.002
	IL2_STAT5_SIGNALING	-1.58	0.002	0.007
	COMPLEMENT	-1.56	<0.001	0.009
	UV_RESPONSE_DN	-1.53	<0.001	0.011
	MITOTIC_SPINDLE	-1.50	0.002	0.013
	APICAL_SURFACE	-1.46	0.033	0.019
	ALLOGRAFT_REJECTION	-1.43	0.008	0.026
	HEDGEHOG_SIGNALING	-1.40	0.054	0.030
Reactome (curated pathway members)	INTERFERON_GAMMA_SIGNALING	-2.24	<0.001	<0.001
	INTERFERON_SIGNALING	-2.14	<0.001	<0.001
	TRAF6_MEDIATED_IRF7_ACTIVATION	-2.08	<0.001	0.001
	CYTOKINE_SIGNALING_IN_IMMUNE_SYSTEM	-2.07	<0.001	0.001
	RIG_I_MDA5_MEDIATED_INDUCTION_OF_IFN_ALPHA_BETA_PATHWAYS	-2.02	<0.001	0.002
	FACTORS_INVOLVED_IN_MEGAKARYOCYTE_DEVELOPMENT_AND_PLATELET_PRODUCTION	-2.01	<0.001	0.002
	CIRCADIAN_CLOCK	-2.00	<0.001	0.002
	NEGATIVE_REGULATORS_OF_RIG_I_MDA5_SIGNALING	-1.95	<0.001	0.004
	BMAL1_CLOCK_NPAS2_ACTIVATES_CIRCADIAN_EXPRESSION	-1.92	<0.001	0.007
	DOWNSTREAM_SIGNAL_TRANSDUCTION	-1.91	<0.001	0.007
	CIRCADIAN_REPRESSION_OF_EXPRESSION_BY_REV_ERBA	-1.88	<0.001	0.009
	INTERFERON_ALPHA_BETA_SIGNALING	-1.87	<0.001	0.011
	YAP1_AND_WWTR1_TAZ_STIMULATED_GENE_EXPRESSION	-1.87	<0.001	0.010
	NOTCH1_INTRACELLULAR_DOMAIN_REGULATES_TRANSCRIPTION	-1.86	<0.001	0.011
	RORA_ACTIVATES_CIRCADIAN_EXPRESSION	-1.83	0.002	0.016
	SIGNALING_BY_FGFR_IN_DISEASE	-1.81	<0.001	0.020
	MEIOTIC_RECOMBINATION	-1.79	<0.001	0.021
	SIGNALING_BY_RHO_GTPASES	-1.78	<0.001	0.022
	PI3K_AKT_ACTIVATION	-1.78	0.006	0.021
	PI3K_EVENTS_IN_ERBB4_SIGNALING	-1.77	0.002	0.024
Signaling pathway targets** (MSigDB, C6 category)	KRAS.300_UP.V1_UP	-1.90	<0.001	0.001
	STK33_NOMO_UP	-1.85	<0.001	0.002
	TGFB_UP.V1_UP	-1.85	<0.001	0.002
	KRAS.600.LUNG.BREAST_UP.V1_UP	-1.81	<0.001	0.002
	KRAS.600_UP.V1_UP	-1.81	<0.001	0.002
	STK33_UP	-1.79	<0.001	0.001
	ESC_V6.5_UP_EARLY.V1_UP	-1.67	<0.001	0.006

		KRAS.LUNG.BREAST_UP.V1_UP	-1.66	<0.001	0.006
		STK33_SKM_UP	-1.63	<0.001	0.008
		SINGH_KRAS_DEPENDENCY_SIGNATURE_	-1.60	0.018	0.009
uXBP1	Hallmark (curated transcriptional targets)	EPITHELIAL_MESENCHYMAL_TRANSITION	2.38	<0.001	<0.001
		MTORC1_SIGNALING	2.15	<0.001	<0.001
		UNFOLDED_PROTEIN_RESPONSE	1.99	<0.001	<0.001
		PROTEIN_SECRETION	1.99	<0.001	<0.001
		OXIDATIVE_PHOSPHORYLATION	1.84	<0.001	0.001
		HYPOXIA	1.83	<0.001	0.001
		GLYCOLYSIS	1.79	<0.001	0.002
		FATTY_ACID_METABOLISM	1.76	<0.001	0.002
		MYOGENESIS	1.71	<0.001	0.003
		MYC_TARGETS_V2	1.69	0.002	0.003
		ANGIOGENESIS	1.67	0.006	0.003
		MYC_TARGETS_V1	1.66	<0.001	0.003
		APICAL_JUNCTION	1.61	<0.001	0.005
		XENOBIOTIC_METABOLISM	1.59	<0.001	0.006
		UV_RESPONSE_DN	1.58	0.004	0.006
		REACTIVE_OXIGEN_SPECIES_PATHWAY	1.56	0.014	0.007
		ADIPOGENESIS	1.55	<0.001	0.007
		CHOLESTEROL_HOMEOSTASIS	1.49	0.008	0.013
	Reactome (curated pathway members)	COLLAGEN_FORMATION	2.59	<0.001	<0.001
		ACTIVATION_OF_CHAPERONE_GENES_BY_XBP1S	2.21	<0.001	<0.001
		SPHINGOLIPID_METABOLISM	2.18	<0.001	<0.001
		METABOLISM_OF_PROTEINS	2.15	<0.001	<0.001
		EXTRACELLULAR_MATRIX_ORGANIZATION	2.14	<0.001	<0.001
		UNFOLDED_PROTEIN_RESPONSE	2.06	<0.001	0.001
		TRANSLATION	2.04	<0.001	0.001
		ASPARAGINE_N_LINKED_GLYCOSYLATION	2.04	<0.001	0.001
		POST_TRANSLATIONAL_PROTEIN_MODIFICATION	2.02	<0.001	0.002
		SPHINGOLIPID_DE_NOVO_BIOSYNTHESIS	1.98	0.002	0.003
		SRP_DEPENDENT_COTRANSLATIONAL_PROTEIN_TARGETING_TO_MEMBRANE	1.96	<0.001	0.004
		GLYCOSPHINGOLIPID_METABOLISM	1.93	0.002	0.006
		LYSOSOME_VESICLE_BIOGENESIS	1.84	0.002	0.020
		MEMBRANE_TRAFFICKING	1.84	<0.001	0.019
		RESPONSE_TO_ELEVATED_PLATELET_CYTOSOLIC_CA2_	1.84	<0.001	0.018
		TRANS_GOLGI_NETWORK_VESICLE_BUDDING	1.79	<0.001	0.032

		ZINC_TRANSPORTERS	1.79	0.004	0.032
		DIABETES_PATHWAYS	1.78	<0.001	0.030
		PHOSPHOLIPID_METABOLISM	1.78	<0.001	0.030
		TRANSFERRIN_ENDOCYTOSIS_AND_RECYCLING	1.78	<0.001	0.029
	Signaling pathway targets** (MSigDB, C6 category)	CORDENONSI_YAP_CONSERVED_SIGNATURE	1.75	0.004	0.022
		ERB2_UP.V1_UP	1.67	<0.001	0.018
		NFE2L2.V2	1.57	<0.001	0.034
		MTOR_UP.N4.V1_UP	1.54	0.004	0.037
		SIRNA_EIF4GI_UP	1.54	0.004	0.035
		ESC_J1_UP_LATE.V1_UP	1.51	<0.001	0.035
		CAMP_UP.V1_UP	1.48	<0.001	0.032
Control (vs. uXBP1)	Hallmark (curated transcriptional targets)	INTERFERON_ALPHA_RESPONSE	-2.47	<0.001	<0.001
		TNFA_SIGNALING_VIA_NFKB	-2.41	<0.001	<0.001
		INTERFERON_GAMMA_RESPONSE	-2.25	<0.001	<0.001
		KRAS_SIGNALING_UP	-2.03	<0.001	<0.001
		INFLAMMATORY_RESPONSE	-1.87	<0.001	0.001
		TGF_BETA_SIGNALING	-1.72	0.002	0.003
		P53_PATHWAY	-1.68	<0.001	0.003
		APOPTOSIS	-1.65	<0.001	0.004
		COMPLEMENT	-1.54	<0.001	0.011
		IL6_JAK_STAT3_SIGNALING	-1.54	0.008	0.010
		ALLOGRAFT_REJECTION	-1.51	0.004	0.013
		WNT_BETA_CATENIN_SIGNALING	-1.44	0.021	0.026
	Reactome (curated pathway members)	INTERFERON_GAMMA_SIGNALING	-2.21	<0.001	<0.001
		CYTOKINE_SIGNALING_IN_IMMUNE_SYSTEM	-2.11	<0.001	0.002
		INTERFERON_SIGNALING	-2.09	<0.001	0.002
		PACKAGING_OF_TELOMERE_ENDS	-2.04	<0.001	0.003
		RNA_POL_I_TRANSCRIPTION	-2.04	<0.001	0.003
		DEPOSITION_OF_NEW_CENPA_CONTAINING_NUCLEOSOMES_AT_THE_CENTROMERE	-2.02	<0.001	0.003
		RNA_POL_I_PROMOTER_OPENING	-2.01	<0.001	0.003
		MEIOTIC_RECOMBINATION	-1.98	<0.001	0.004
		TRAF6_MEDIATED_IRF7_ACTIVATION	-1.93	<0.001	0.007
		AMYLOIDS	-1.90	<0.001	0.011
		FACTORS_INVOLVED_IN_MEGAKARYOCYTE_DEVELOPMENT_AND_PLATELET_PRODUCTION	-1.89	<0.001	0.011
		INTERFERON_ALPHA_BETA_SIGNALING	-1.86	<0.001	0.015
		CIRCADIAN_REPRESSION_OF_EXPRESSION_BY_REV_ERBA	-1.85	<0.001	0.017

	MEIOSIS	-1.84	<0.001	0.017
	RNA_POL_I_RNA_POL_III_AND_MITOCHONDRIAL_TRANSCRIPTION	-1.82	<0.001	0.020
	MEIOTIC_SYNAPSIS	-1.81	<0.001	0.020
	NUCLEOTIDE_BINDING_DOMAIN_LEUCINE_RICH_REPEAT_CONTAINING_RECEPTOR_NLR_SIGNALING_PATHWAYS	-1.79	<0.001	0.023
	BMAL1_CLOCK_NPAS2_ACTIVATES_CIRCADIAN_EXPRESSION	-1.78	<0.001	0.025
	NUCLEAR_SIGNALING_BY_ERBB4	-1.78	0.004	0.025
	TRANSCRIPTIONAL_REGULATION_OF_WHITE_ADIPOCYTE_DIFFERENTIATION	-1.78	0.002	0.024
Transcription factor targets*	IRF_Q6	-1.95	<0.001	0.001
	ISRE_01	-1.86	<0.001	0.009
	NFKB_Q6_01	-1.77	<0.001	0.017
	PR_02	-1.76	<0.001	0.014
	MZF1_02	-1.74	<0.001	0.014
	ICSBP_Q6	-1.73	<0.001	0.013
	AR_03	-1.72	0.004	0.013
	NFKB_C	-1.69	<0.001	0.019
	STTTCRNTTT_IRF_Q6	-1.68	<0.001	0.020
	NFKB_Q6	-1.68	<0.001	0.019
	CDP_01	-1.65	<0.001	0.022
	NGFIC_01	-1.64	<0.001	0.023
	NFKAPPAB65_01	-1.63	<0.001	0.023
	CEBP_C	-1.61	<0.001	0.026
	ETF_Q6	-1.61	<0.001	0.025
	IRF2_01	-1.59	<0.001	0.028
Signaling pathway targets** (MSigDB, C6 category)	KRAS.600.LUNG.BREAST_UP.V1_UP	-2.05	<0.001	<0.001
	KRAS.300_UP.V1_UP	-2.04	<0.001	<0.001
	KRAS.LUNG.BREAST_UP.V1_UP	-2.00	<0.001	<0.001
	STK33_NOMO_UP	-1.84	<0.001	0.002
	KRAS.600_UP.V1_UP	-1.81	<0.001	0.002
	STK33_UP	-1.79	<0.001	0.002
	KRAS.BREAST_UP.V1_UP	-1.77	<0.001	0.003
	KRAS.LUNG_UP.V1_UP	-1.71	<0.001	0.004
	KRAS.DF.V1_UP	-1.65	<0.001	0.007
	KRAS.50_UP.V1_UP	-1.64	0.002	0.007
	STK33_SKM_UP	-1.56	0.002	0.017
	SINGH_KRAS_DEPENDENCY_SIGNATURE_	-1.56	0.032	0.016

CSR_EARLY_UP.V1_UP	-1.51	<0.001	0.025

*Sequence motifs with known transcription factor are shown.
**Upregulated target gene sets in response to pathway activation are shown.
FDR: false discovery rate, NES: normalized enrichment score.
Gene sets were obtained from Molecular Signature Database (MSigDB) (www.broadinstitute.org/msigdb).

Supplementary Table 4

XBP1 target gene signature.

Overexpressed in	Entrez Gene ID	Gene symbol
sXBP1	10954	PDIA5
	51569	UFM1
	151887	CCDC80
	55829	SELS
	29100	TMEM208
	1346	COX7A1
	115701	ALPK2
	56922	MCCC1
	55076	TMEM45A
	90411	MCFD2
	164781	WDR69
	8544	PIR
	1300	COL10A1
	100128892	LOC100128892
	728495	FAM74A3
	10058	ABCB6
	93953	ACRC
	55974	RAG1AP1
	160428	ALDH1L2
	1013	CDH15
	55605	KIF21A
	3486	IGFBP3
	9871	SEC24D
	10897	YIF1A
	10717	AP4B1
	590	BCHE
	58515	SELK
	29100	HSPC171
	2053	EPHX2
	4015	LOX
	79947	DHDDS

Overexpressed in	Entrez Gene ID	Gene symbol
Control (vs. sXBP1)	1906	EDN1
	6876	TAGLN
	5327	PLAT
	441951	C20orf199
	10855	HPSE
	400759	LOC400759
	25976	TIPARP
	153222	C5orf41
	1958	EGR1
	5328	PLAU
	4487	MSX1
	10019	SH2B3
	55450	CAMK2N1
	125050	RN7SK
	1316	KLF6
	2633	GBP1
	1848	DUSP6
	56978	PRDM8
	9518	GDF15
	114801	TMEM200A
	2114	ETS2
	55711	FAR2
	5764	PTN
	114548	NLRP3
	730249	IRG1
	100134073	LOC100134073
	1839	HBEGF
	282618	IL29
	730249	LOC730249
	100133034	LOC100133034
	84941	HSH2D

401508	FLJ45202	6373	CXCL11
55840	EAF2	115361	GBP4
56521	DNAJC12	51191	HERC5
255520	ELMOD2	3456	IFNB1
100131785	LOC100131785	4061	LY6E
728324	LOC728324	116372	LYPD1
121457	IKBIP	93082	LINCR
1842	ECM2	8911	CACNA1I
1289	COL5A1	57664	PLEKHA4
645345	CHCHD9	9353	SLIT2
4189	DNAJB9	259307	IL4I1
11153	FICD	3627	CXCL10
79586	CHPF	118429	ANTXR2
6723	SRM	51251	NT5C3
57535	KIAA1324	2621	GAS6
79174	CRELD2	23645	PPP1R15A
51009	DERL2	834	CASP1
64116	SLC39A8	2828	GPR4
5226	PGD	8743	TNFSF10
202459	OSTCL	344148	NCKAP5
22998	LIMCH1	7185	TRAF1
6038	RNASE4	142679	DUSP19
90522	YIF1B	10468	FST
55033	FKBP14	220	ALDH1A3
1277	COL1A1	54979	HRASLS2
84513	PPAPDC1B	3142	HLX
9060	PAPSS2	81030	ZBP1
5961	PRPH2	114793	FMNL2
2330	FMO5	4321	MMP12
646567	LOC646567	643427	RBMS2P
27090	ST6GALNAC4	92241	RCSD1
654174	LOC654174	3790	KCNS3
51651	PTRH2	11082	ESM1
10300	KATNB1	3487	IGFBP4
157317	CYCSL1	2040	STOM

	283	ANG
	80328	ULBP2
	58477	SRPRB
	10609	SC65
	9424	KCNK6
	6748	SSR4
	5238	PGM3
	23753	SDF2L1
	10567	RABAC1
	340542	BEX5
	51726	DNAJB11
	55002	TMCO3
	29995	LMCD1
	10525	HYOU1
	81031	SLC2A10
	90701	SEC11C
	51136	RNFT1
	100134537	LOC100134537
	26003	GORASP2
	57016	AKR1B10
	343477	TRA1P2
	378	ARF4
	10113	PREB
	3703	STT3A
	84561	SLC12A8
	11015	KDELR3
	516	ATP5G1
	7466	WFS1
	58505	OSTC
	138162	C9orf116
	55741	EDEM2
	7184	HSP90B1
	7873	ARMET
	10263	CDK2AP2
uXBP1	6447	SCG5

	6352	CCL5
	80176	SPSB1
	23387	SIK3
	6349	CCL3L1
	3087	HHEX
	5939	RBMS2
	10544	PROCR
	7508	XPC
	4005	LMO2
	11221	DUSP10
	114915	NCRNA00219
	3732	CD82
	135112	NCOA7
	290	ANPEP
	7980	TFPI2
	50650	ARHGEF3
	80019	UBTD1
	623	BDKRB1
	414062	CCL3L3
	4314	MMP3
	79971	GPR177
	11033	CENTA1
	8325	FZD8
	149111	CNIH3
	2697	GJA1
	8877	SPHK1
	10253	SPRY2
	55691	FRMD4A
	81622	UNC93B1
	3134	HLA-F
	10437	IFI30
	22822	PHLDA1
	8334	HIST1H2AC
	595	CCND1
Control (vs. uXBP1)	27128	PSCD4

54928	IMPAD1	9076	CLDN1
284029	C17orf44	10855	HPSE
84624	FNDC1	1839	HBEGF
2202	EFEMP1	10113	PREB
126393	HSPB6	400759	LOC400759
64798	DEPDC6	92241	RCSD1
79751	SLC25A22	441951	C20orf199
9645	MICAL2	1440	CSF3
160428	ALDH1L2	730249	IRG1
10263	CDK2AP2	3866	KRT15
9114	ATP6V0D1	282618	IL29
10516	FBLN5	730249	LOC730249
5021	OXTR	3598	IL13RA2
202459	OSTCL	9518	GDF15
10272	FSTL3	7185	TRAF1
654174	LOC654174	93082	LINCR
10058	ABCB6	6373	CXCL11
340542	BEX5	84941	HSH2D
8076	MFAP5	89795	NAV3
338773	TMEM119	4321	MMP12
166929	SGMS2	1316	KLF6
9531	BAG3	1848	DUSP6
8544	PIR	220	ALDH1A3
1404	HAPLN1	54979	HRASLS2
7873	ARMET	7980	TFPI2
4642	MYO1D	5328	PLAU
22801	ITGA11	10019	SH2B3
55076	TMEM45A	11082	ESM1
84898	PLXDC2	8911	CACNA1I
100134537	LOC100134537	100133034	LOC100133034
646567	LOC646567	10060	ABCC9
58515	SELK	27128	CYTH4
1490	CTGF	6352	CCL5
2191	FAP	330	BIRC3
10924	SMPDL3A	4487	MSX1

54575	UGT1A10	56978	PRDM8
389336	C5orf46	51251	NT5C3
57214	KIAA1199	25976	TIPARP
728324	LOC728324	3456	IFNB1
1842	ECM2	441525	SPANXN4
7373	COL14A1	4314	MMP3
26003	GORASP2	142679	DUSP19
6356	CCL11	2633	GBP1
1300	COL10A1	2828	GPR4
871	SERPINH1	2330	FMO5
343477	TRA1P2	352999	C6orf58
10717	AP4B1	23645	PPP1R15A
56521	DNAJC12	153222	C5orf41
121457	IKBIP	81030	ZBP1
10897	YIF1A	8743	TNFSF10
6876	TAGLN	114793	FMNL2
64175	LEPRE1	6781	STC1
79174	CRELD2	8325	FZD8
4015	LOX	5764	PTN
151887	CCDC80	10253	SPRY2
100128892	LOC100128892	2114	ETS2
23753	SDF2L1	623	BDKRB1
90522	YIF1B	259307	IL4I1
728495	FAM74A3	8942	KYNU
157317	CYCSL1	90865	IL33
57016	AKR1B10	3087	HHEX
115701	ALPK2	135112	NCOA7
55840	EAF2	79670	ZCCHC6
5226	PGD	11009	IL24
3486	IGFBP3	114548	NLRP3
9424	KCNK6	1958	EGR1
58477	SRPRB	10437	IFI30
9060	PAPSS2	3656	IRAK2
1277	COL1A1	27075	TSPAN13
84886	C1orf198	3627	CXCL10

590	BCHE	134285	TMEM171
3703	STT3A	22822	PHLDA1
55605	KIF21A	3142	HLX
51726	DNAJB11	643427	RBMS2P
6038	RNASE4	3620	INDO
1289	COL5A1	115361	GBP4
378	ARF4	30014	SPANXA1
100131785	LOC100131785	10903	MTMR11
9915	ARNT2	3620	IDO1
283	ANG	100133171	LOC100133171
64116	SLC39A8	5743	PTGS2
58505	OSTC	55711	FAR2
10525	HYOU1	80176	SPSB1
27090	ST6GALNAC4	8767	RIPK2
9891	NUAK1	8334	HIST1H2AC
645345	CHCHD9	4005	LMO2
10609	SC65	220323	OAF
84561	SLC12A8	290	ANPEP
29995	LMCD1	7128	TNFAIP3
10300	KATNB1	6948	TCN2
401508	FLJ45202	11174	ADAMTS6
57535	KIAA1324	2635	GBP3
138162	C9orf116	3732	CD82
55002	TMCO3	27284	SULT1B1
22998	LIMCH1	629	CFB
11015	KDELR3	57664	PLEKHA4
7466	WFS1	118429	ANTXR2
51136	RNFT1	6354	CCL7
7184	HSP90B1	441024	MTHFD2L

Supplementary Table 5

Transcriptome datasets used for assessment of XBP1 target gene signatures.

Species	Intervention/disease	Sample type	Accession number*
Mouse	Carbon tetrachloride (CCl4)	Primary HSC	GSE34640
	Bile duct ligation (BDL)	Primary HSC	GSE34640
	Culture activated	Primary HSC	GSE34640
Mouse	High fat diet (HFD) with cholesterol	Tissue	GSE40481
Human	Healthy obese, NAFLD, NASH	Tissue	GSE61260
	Advanced NASH	Tissue	GSE49541

*NCBI Gene Expression Omnibus (GEO) database accession number.

HSC: hepatic stellate cell.
